# 64 Conical teeth? Think outside the box!

**DOI:** 10.1093/rheumatology/keac496.060

**Published:** 2022-10-07

**Authors:** N Boutrid, H Rahmoune, B Bioud, M Amrane

**Affiliations:** 1 Pediatrics, Sétif University Hospital, Algeria; 2 LMCVGN research laboratory, Sétif 1 University, Algeria

## Abstract

**Background:**

Ectodermal dysplasias (ED) are inherited disorders involving congenital abnormalities of different ectodermal structures, the most prominent presentation being adontia/hypodontia

In this article, we present a brief overview of several pediatric cases of ED with a short review of the odontological manifestations.

**2. Case reports:**

We report several cases of children consulting in the pediatric department for abnormal teeth, with or without systemic manifestations.

The clinical examination of these patients revealed insufficient and abnormal dentition, with rare beveled teeth and thin and rare hair, as well as eyelashes and eyebrows.

Also, parents reported episodes of hyperthermia without sweating from an early age.

Dental radiography confirmed the diagnosis of ectodermal dysplasia.

**3. Discussion:**

Ectodermal dysplasias (EDs) are a group of various inherited disorders involving abnormal congenital development of at least two ectodermal structures (hair, nails, teeth, and sweat glands). The management of these rare disabling conditions is still mainly symptomatic. The traditional removable prosthesis can be an option to replace missing teeth. However, implants provide the best long-term results and prognosis. In particular, dental implants are commonly used in oral reconstruction of ED patients, but long-term data on bone augmentation and bone resorption, aesthetic outcomes, and implant success are needed.

**4. Conclusion:**

EDs are a myriad of heterogeneous conditions encompassing several inherited embryopathies affecting teeth and other ectoderm-derived structures *in utero*.

Diagnosis is essentially clinical, confirmed by radiology and genetics which can specify the causal mutation; treatment is mainly conservative.

